# Primary Aldosteronism: Metabolic Reprogramming and the Pathogenesis of Aldosterone-Producing Adenomas

**DOI:** 10.3390/cancers13153716

**Published:** 2021-07-23

**Authors:** Siyuan Gong, Martina Tetti, Martin Reincke, Tracy Ann Williams

**Affiliations:** 1 Medizinische Klinik und Poliklinik IV, Klinikum der Universität München, Ludwig-Maximilians-Universität München, 80336 Munich, Germany; Siyuan.Gong@med.uni-muenchen.de (S.G.); martina.tetti@med.uni-muenchen.de (M.T.); Martin.Reincke@med.uni-muenchen.de (M.R.); 2 Department of Medical Sciences, Division of Internal Medicine and Hypertension, University of Turin, 10126 Turin, Italy

**Keywords:** adaptive metabolism, adrenal gland, conn adenoma, fatty acid metabolism, ferroptosis, hyperaldosteronism, metabolic reprogramming, β-oxidation, PPARα, tumor microenvironment

## Abstract

**Simple Summary:**

Primary aldosteronism is a common form of endocrine hypertension often caused by a hyper-secreting tumor of the adrenal cortex called an aldosterone-producing adenoma. Metabolic reprogramming plays a role in tumor progression and influences the tumor immune microenvironment by limiting immune-cell infiltration and suppressing its anti-tumor function. We hypothesized that the development of aldosterone-producing adenomas involves metabolic adaptations of its component tumor cells and intrinsically influences tumor pathogenesis. Herein, we use state-of-the-art computational tools for the comprehensive analysis of array-based gene expression profiles to demonstrate metabolic reprogramming and remodeling of the immune microenvironment in aldosterone-producing adenomas compared with paired adjacent adrenal cortical tissue. Our findings suggest metabolic alterations may function in the pathogenesis of aldosterone-producing adenomas by conferring survival advantages to their component tumor cells.

**Abstract:**

Aldosterone-producing adenomas (APAs) are characterized by aldosterone hypersecretion and deregulated adrenocortical cell growth. Increased energy consumption required to maintain cellular tumorigenic properties triggers metabolic alterations that shape the tumor microenvironment to acquire necessary nutrients, yet our knowledge of this adaptation in APAs is limited. Here, we investigated adrenocortical cell-intrinsic metabolism and the tumor immune microenvironment of APAs and their potential roles in mediating aldosterone production and growth of adrenocortical cells. Using multiple advanced bioinformatics methods, we analyzed gene expression datasets to generate distinct metabolic and immune cell profiles of APAs versus paired adjacent cortex. APAs displayed activation of lipid metabolism, especially fatty acid β-oxidation regulated by PPARα, and glycolysis. We identified an immunosuppressive microenvironment in APAs, with reduced infiltration of CD45^+^ immune cells compared with adjacent cortex, validated by CD45 immunohistochemistry (3.45-fold, *p* < 0.001). APAs also displayed an association of lipid metabolism with ferroptosis and upregulation of antioxidant systems. In conclusion, APAs exhibit metabolic reprogramming towards fatty acid β-oxidation and glycolysis. Increased lipid metabolism via PPARα may serve as a key mechanism to modulate lipid peroxidation, a hallmark of regulated cell death by ferroptosis. These findings highlight survival advantages for APA tumor cells with metabolic reprogramming properties.

## 1. Introduction

Primary aldosteronism (PA) is the most frequent secondary cause of hypertension characterized by the overproduction of aldosterone relatively autonomous of the renin-angiotensin system. PA is generally classified into unilateral and bilateral forms, which determine the surgical or pharmacological treatment of the disease [1]. The surgical management of unilateral PA has made available, as a side effect, tissue sample specimens for a wide range of scientific studies. Histopathology shows that the surgically removed adrenals mainly display an aldosterone-producing adenoma (APA) with somatic mutations in a few genes that cause constitutive aldosterone production [2,3,4,5]. The variants usually occur in genes that encode ion channels or ATPases and function in the regulation of cellular ion homeostasis [6]. Of these, the KCNJ5 inwardly rectifying potassium channel (also called GIRK4) displays the highest prevalence of variants in most reported populations. Furthermore, in vivo observations and in vitro findings suggest that KCNJ5 mutations are likely to also cause cell proliferation [7,8,9]. The role of somatic mutations in constitutive aldosterone production is well defined, but many other mechanisms may modulate aldosterone production from APAs [10,11,12,13,14,15]. Regulated forms of cell death, including apoptosis [16] and ferroptosis [17], have also been implicated in the pathogenesis of APAs. 

Metabolic reprogramming has a well-characterized role in cancer progression [18]. Increasing evidence suggests that tumor cells must modify their metabolism in response to their elevated energy requirements [19], and metabolic adaptations have been described in many types of cancers [20,21,22]. Of note, tumor cells undergo metabolic reprogramming that may modify the tumor microenvironment (TME) to fulfill the demands of biosynthesis and growth [23]. Tumor-infiltrating immune cells also rely on nutrients in the TME, and metabolic competition between tumor cells and infiltrating immune cells hamper or eliminate the anti-tumor immune response [23,24]. Furthermore, the high metabolic activity of tumor cells can generate metabolites (e.g., adenosine, kynurenine, and acidosis) that may accumulate to toxic concentrations, target immune suppressive cells and inhibit their function [25,26,27]. For instance, increased glycolysis in cancer cells (the Warburg effect) produces lactate that acidifies the TME and interferes with immune-cell effector function [28]. 

Because APAs are hormone-producing adenomas, an increased metabolic demand compared with adjacent tissue would be expected to sustain aldosterone hypersecretion. In this study, we investigated metabolic differences between APAs and paired adjacent cortical tissue to investigate mechanisms of the TME to support the development and progression of an APA.

## 2. Materials and Methods

### 2.1. Data Preprocessing

We analyzed microarray gene expression data from GSE64957 [29] and GSE60042 [30] from the Gene Expression Omnibus (GEO) database (https://www.ncbi.nlm.nih.gov/geo/ access date for GSE64957 and GSE60042: 1 January 2021 and 5 April 2021, respectively). GSE64957 comprised a data set from 13 APAs with corresponding paired zona glomerulosa, and zona fasciculata samples (7 APAs with a *KCNJ5* mutation and 6 APAs with no mutation detected). GSE60042 comprised 7 APAs and paired adjacent adrenal cortex tissue samples. GSE64957 Affymetrix microarray raw data were processed using the robust multichip average (RMA) algorithm with R package oligo for background adjustment, quantile normalization, log-transformation, and Combat function of R package sva (surrogate variable analysis) was used for batch correction. The expression matrix of GSE60042 was extracted from series matrix files downloaded from the GEO database using the GEOquery package, followed by standardization using the normalize Between Arrays function of the limma R package. The gene expression datasets were translated into commonly used gene symbols for further analyses.

The R package “limma” was used to clarify differentially expressed genes among paired groups; differentially expressed genes with adjust *p* < 0.05 and log_2_ fold change (FC) (log_2_FC) >1 were selected for further functional enrichments.

### 2.2. Patient Samples

Resected adrenal samples for histology and immunohistochemistry analyses were from patients diagnosed with unilateral primary aldosteronism following European Society of Hypertension guidelines [31,32] at the Medizinische Klinik IV, Klinikum Ludwig-Maximilians-Universität München, Munich, Germany, in accordance with local criteria for adrenal venous sampling [33]. These APAs comprised 6 with a *KCNJ5* mutation and 6 without *KCNJ5* mutations (2 *CACNA1D*, 2 *ATP1A1*, and 2 with no mutation detected). These patients gave written informed consent for use of biomaterial for medical research in accordance with the local ethics committee.

### 2.3. Functional Enrichments

To identify biological processes and pathway enrichment associated with differentially expressed genes, we used the gene set enrichment analysis (GSEA) method based on Gene Ontology (GO), Kyoto Encyclopedia of Genes and Genomes (KEGG), HALLMARK and Reactome gene sets from MSigDB database with the clusterProfiler package of R. The online tools of Metascape^®^ (https://metascape.org access data: 11 April 2021) [34] and g:profiler (https://biit.cs.ut.ee/gprofiler access data: 21 April 2021) [35] were also used to identify pathway interactions, and protein–protein interaction networks, and to comprehensively understand the biology of differentially expressed genes using different independent knowledge bases (e.g., WikiPathways) to summarize the function of identified genes. 

### 2.4. Determination of Tumor Immune Microenvironment and Immune Cell Infiltration Patterns

To assess tumor immune microenvironments, the “Estimation of Stromal and Immune cells in Malignant Tumors using Expression data” (ESTIMATE) algorithm [36] was used to quantify the infiltrating immune cell level (immune score) and stromal content (stromal score) for each sample, using gene expression signatures.

To further evaluate the immune characteristics of APA and adjacent zona glomerulosa cells, single sample GSEA (ssGSEA) [37] analysis was performed to identify the relative proportions of 28 immune cell types in the TME based on the feature gene panels for each immune cell type [38,39]. In addition, the Microenvironment Cell Populations-counter (MCP-counter) algorithm [40,41] was used to calculate stromal cell abundance, including endothelial cells and cancer-associated fibroblasts.

### 2.5. Identification of Ferroptosis-Related, Immune-Related, and Reactive Oxygen Species (ROS)-Related Genes

The corresponding ferroptosis-related gene list was downloaded from FerrDb [42]. In total, we identified 259 ferroptosis-related genes, including 108 drivers, 69 suppressors, and 111 markers. The immune-related gene lists were obtained from ImmPort (https://www.immport.org/resources access data: 21 December 2020). The ROS-related gene list was collected from the GeneCards database (https://www.genecards.org/ access data: 4 April 2021) using the term “reactive oxygen species”, and only genes with a relevance score >7 were considered. All significantly differentially expressed genes were set at adjust *p*-value < 0.05 and |log_2_FC| > 1.

### 2.6. Immunohistochemistry

Formalin fixed paraffin-embedded sections of APA tissue were incubated with anti-CD45 primary antibody (#13917; Cell Signaling Technology, Danvers, MA, USA) at 4 °C overnight. Immunohistochemistry staining was performed using ZytoChem Plus HRP Polymer kit (Zytomed, Berlin, Germany) following the manufacturer’s instructions and quantified with QuPath (v.0.2.3, University of Edinburgh, Edinburgh, UK) using the positive cell detection feature with empirical parameters.

### 2.7. Cell Line and Culture Conditions

Human adrenocortical (HAC15) cells (a kind gift from Professor William E. Rainey, University of Michigan, Ann Arbor, MI, USA) were cultured in Dulbecco’s Modified Eagle/F12 medium with L-glutamine containing 10% (*v/v*) cosmic calf serum, 1% antibiotic-antimycotic, 1% insulin-transferrin-selenium, and 50 mg/mL gentamycin at 37 °C and 5% CO_2_.

### 2.8. Cell Viability Assay

HAC15 cells (4 × 10^4^ per well) were seeded on 96-well plates for 24 h and then treated with etomoxir (E1905, Sigma-Aldrich, St.Louis, MO, USA). Cell viability was measured using WST-1 assay according to the manufacturer’s instructions (Roche Diagnostics GmbH, Mannheim, Germany). Cells without etomoxir treatment were used as a control.

### 2.9. Statistics

R software (version 4.0.3, Vienna, Austria) and GraphPad Prism 8.0 (GraphPad Software Inc., San Diego, CA, USA) were employed for figures generation and statistical analyses. Differences between the two groups were analyzed through paired *t*-test or paired Wilcoxon test, whereas Kruskal–Wallis test or One-way ANOVA was performed between groups. The statistically significant level was set as *p* < 0.05.

## 3. Results

### 3.1. Transcriptome Defined Metabolic Reprogramming towards Fatty Acid β-Oxidation and Glycolysis in APAs 

**Figure 1:**
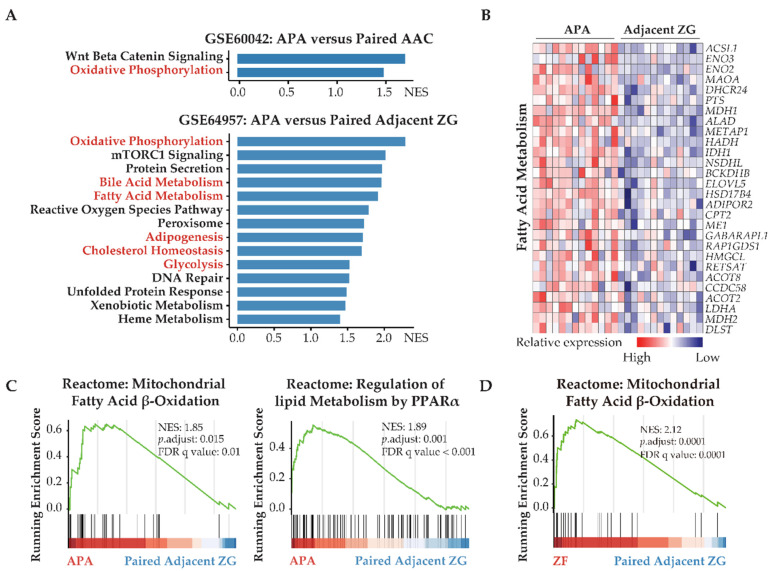
APAs undergo transcriptomic alterations toward increased fatty acid metabolism and glycolysis. (**A**) GSEA showing MSigDB hallmark of upregulated differentially expressed genes in APAs versus AAC (GSE60042) and adjacent ZG (GSE64957). Pathways in red indicate functions related to lipid metabolism and glycolysis. (**B**) Heatmap of the significant upregulated differentially expressed genes with *p* value < 0.05 for fatty acid metabolism in APAs compared with adjacent ZG. (**C**) GSEA plots showing Reactome pathways of lipid biological processes in APAs versus adjacent ZG. (**D**) GSEA plots showing Reactome pathways of mitochondrial fatty acid β-oxidation in ZF versus paired adjacent ZG. APA, aldosterone-producing adenoma; AAC, adjacent adrenal cortex; ZG, zona glomerulosa; ZF, zona fasciculata; NES, normalized enrichment score; FDR, false discovery rate.

Transcriptome data from GSE60042 were used to analyze the biology of differentially expressed genes in APAs versus paired adjacent adrenal cortex. The top upregulated gene sets were related to oxidative phosphorylation (Figure 1A) consistent with a proteomic analysis of APAs [43]. 

**Figure 2:**
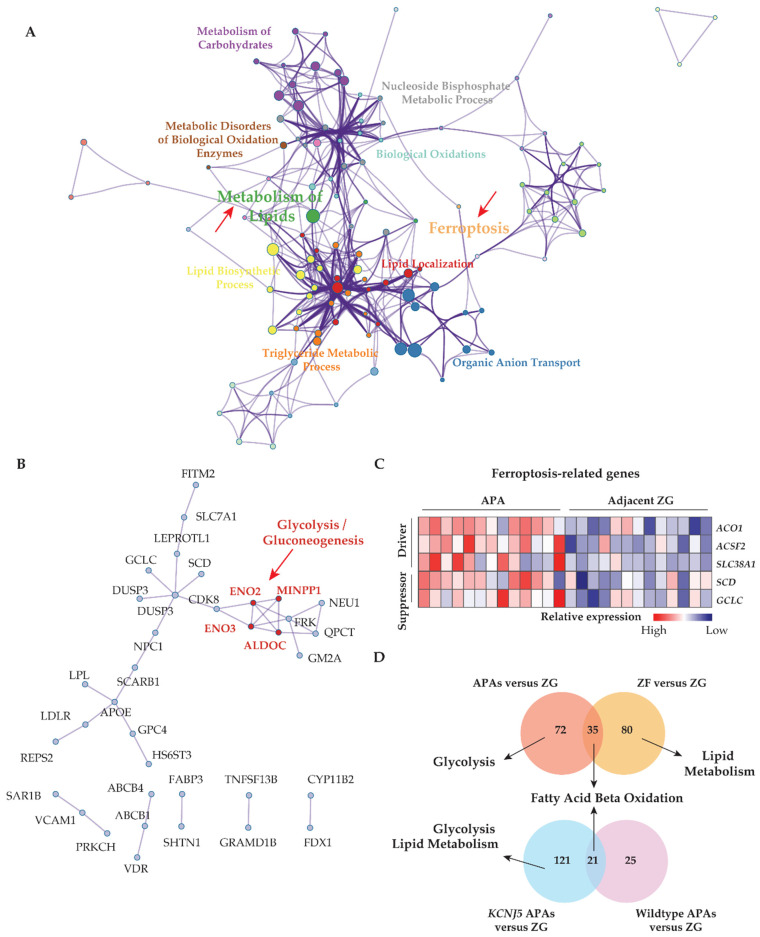
The interaction between lipid metabolism and ferroptosis, and the association of metabolic alterations with adjacent adrenal cortical tissue and APA genotype. (**A**) Metascape functional enrichment analysis of upregulated differentially expressed genes in APAs versus paired adjacent ZG (GSE64957). One node per enriched term, colored by cluster ID. Node size indicates the number of differentially expressed genes involved in the enriched term. (**B**) Protein–protein network using Molecular Complex Detection (MCODE) algorithm. Red fonts represent the core genes of the network involved in the glycolysis/gluconeogenesis pathway. (**C**) Heat map of ferroptosis-related upregulated differentially expressed genes grouped as driver and suppressor in APAs compared paired adjacent ZG. (**D**) Venn diagrams showing the number of unique and overlapping upregulated differentially expressed genes from APAs and adjacent ZF of those APAs to paired adjacent ZG comparisons, and from *KCNJ5* mutated APAs and APAs without mutations (Wild type) to their adjacent ZG comparisons. Top pathways in which the distinct differentially expressed genes from APAs, adjacent ZF, *KCNJ5*-mutated APA, and APA without mutations (Wild type), respectively compared to paired adjacent ZG, using g:profiler online tool. APA, aldosterone-producing adenoma. AAC, adjacent adrenal cortex; ZG, zona glomerulosa; ZF, zona fasciculata; DEGs, differentially expressed genes; NES, normalized enrichment score; FDR, false discovery rate.

Transcriptome data from GSE64957 were used to evaluate potential metabolic differences between APAs and paired adjacent zona glomerulosa. Alterations in transcriptome signatures related to metabolic synthesis were distinguished, with striking gene set enrichments in APAs of oxidative phosphorylation, fatty acid metabolism, and glycolysis (Figure 1A,B). Reactome gene sets demonstrated that the most significantly upregulated signaling pathways in APAs were mitochondrial fatty acid β-oxidation and peroxisome proliferator-activated receptor-α (PPARα) (Figure 1C). These data suggest that APAs may oxidize fatty acids as an energy source for tumor growth and/or steroidogenesis through PPARα signaling. We explored potential crosstalk between these signaling pathways using Metascape analyses with ClueGo, a Cystoscope plug-in. These network analyses highlighted that most signaling pathways involved aspects of lipid biology at the core of the pathogenesis of APAs. Furthermore, we demonstrated a functional link between lipid biology and ferroptosis. This is relevant because adrenocortical cells have previously been shown to be highly sensitive to cell death by ferroptosis due to the inhibition of glutathione biosynthesis (Figure 2A) [17,44,45]. Of note, the ferroptosis suppressor gene coding for glutamate-cysteine ligase catalytic subunit (GCLC) and the gene coding for stearoyl-CoA desaturase (SCD) are highly expressed in APAs compared with adjacent zona glomerulosa (Figure 2C). In addition, protein–protein interaction network analysis of differentially expressed genes revealed an upregulation of hub genes involved in glycolysis/gluconeogenesis using the Molecular Complex Detection algorithm (Figure 2B). Collectively, our evidence indicates that metabolic reprogramming towards fatty acid β-oxidation and glycolysis may confer some metabolic advantage to the APA microenvironment that may sustain tumor cell growth and aldosterone overproduction.

The GSE64957 data set was used to evaluate potential metabolic differences between APAs and paired adjacent zona fasciculata. Fatty acid β-oxidation was observed in adjacent zona fasciculata relative to paired adjacent zona glomerulosa (Figure 1D). We further analyzed the upregulated differentially expressed genes in APAs compared with adjacent zona glomerulosa and in paired zona fasciculata, compared with adjacent zona glomerulosa (Figure 2D). APAs specifically overexpressed genes related to glycolysis/gluconeogenesis, whereas genes that were exclusively upregulated in the paired adjacent zona fasciculata were enriched in pathways related to lipid metabolism. 

### 3.2. KCNJ5 Mutations and Metabolic Reprogramming 

APAs with *KCNJ5* mutations show an increased proliferative index compared with other APAs [9]. We investigated if *KCNJ5* mutated APAs display distinct metabolic features. We showed that genes involved in glycolysis and lipid metabolism displayed enhanced transcription in *KCNJ5* mutated APAs relative to Wild type APAs (both normalized to their adjacent zona glomerulosa) (Figure 2D).

### 3.3. Fatty Acid Oxidation Is Required for the Survival of Human Adrenocortical Cells

**Figure 3:**
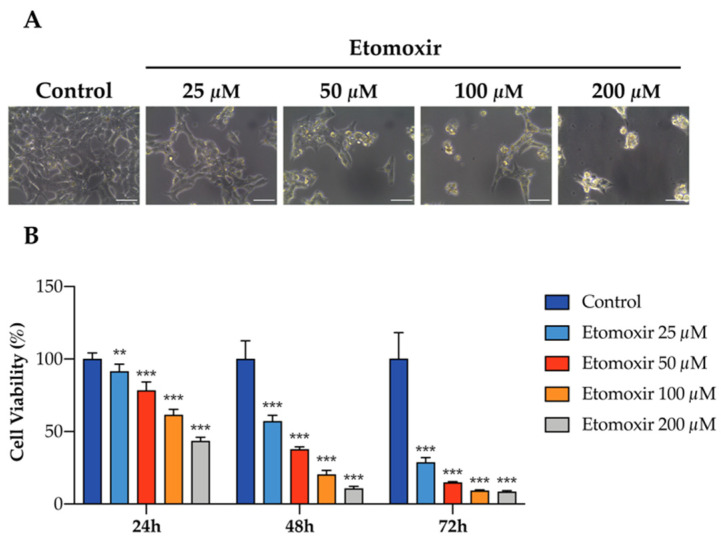
Inhibition of fatty acid oxidation induces cell death in human HAC15 cells. (**A**) Representative images of the effects of etomoxir in HAC15 cells in 6-well plate for 72 h. Scale Bar: 50 µm. (**B**) Cell viability in HAC15 cells treated with increasing concentration of etomoxir (25–200 µM) for 24 h, 48 h, and 72 h. Data are presented as mean ± s.d., One-way ANOVA; ** *p* < 0.01, *** *p* < 0.001.

To further elaborate the functional role of fatty acid oxidation in human adrenal cells, HAC15 cells were treated with etomoxir in culture, an inhibitor of fatty acid oxidation via carnitine palmitoyltransferase-1 inhibition. Etomoxir significantly decreased cell viability of HAC15 cells in a dose-s and time-dependent manner (Figure 3A,B), indicating that fatty acid oxidation may support adrenocortical cell growth. 

### 3.4. Immune Phenotype Alterations in APAs

**Figure 4:**
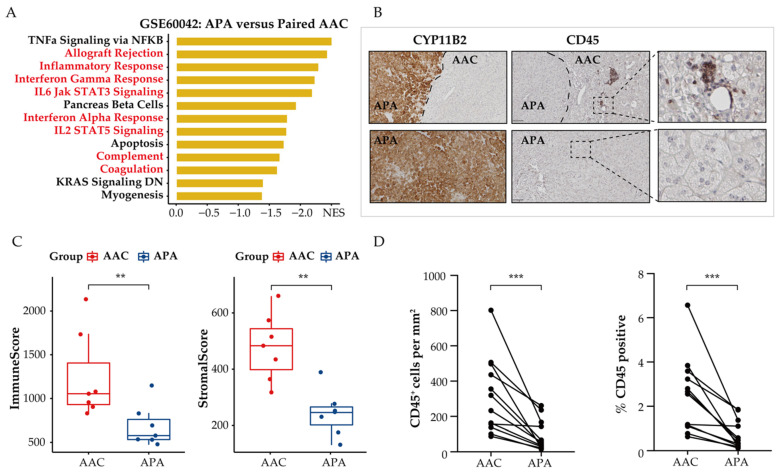
Spatial organization of tumor infiltrating immune cells in APAs versus paired adjacent adrenal cortex (AAC). (**A**) GSEA showing MSigDB hallmark of downregulated differentially expressed genes in APAs versus AAC (GSE60042). Pathways in red indicate functions related to immune response. (**B**) CYP11B2 and CD45 immunohistochemistry staining. The border of APA is defined by the CYP11B2 immunohistochemistry. Scare bar: 100 μm. (**C**) ESTIMATE algorithm showing the distribution of ImmuneScore, StromalScore in APAs versus AAC. ** *p* < 0.01 by paired *t* test. (**D**) CD45^+^ immune cell density and positive percentage between APAs and AAC. *** *p* < 0.001 by paired Wilcox test. APA, aldosterone-producing adenoma; AAC, adjacent adrenal cortex; NES, normalized enrichment score.

Dysregulation of tumor cell metabolism is known to contribute to immune evasion within the TME. Therefore, we investigated the effect of metabolic reprogramming of the TME on tumor-infiltrating immune-cell populations in APAs and adjacent adrenal cortical tissue. We used GSEA to screen for downregulated differentially expressed genes in APAs versus paired adjacent adrenal cortex and identified a vast number of immune-related pathways that were relatively increased in adjacent adrenal cortex, including inflammatory response, interferon-gamma response, and IL6 JAK STAT3 signaling (Figure 4A). Further analysis using the ESTIMATE algorithm to predict immune states revealed a statistically significant decrease of the immune and stromal score in APAs compared with paired adjacent adrenal cortex (*p* < 0.01, paired *t* test, Figure 4C). Collectively, APAs had a higher proportion of tumor cells. To validate these findings, we performed immunohistochemistry analysis of the surface protein CD45, a common marker of immune cells, on 12 formalin-fixed paraffin embedded APA samples with attached adjacent adrenal cortex (Figure 4B). The density and frequency of CD45^+^ cells per mm^2^ were significantly lower in APAs relative to the adjacent adrenal cortex (3.45-fold, *p* < 0.001, paired Wilcoxon test, Figure 4D), which included 6 *KCNJ5*-mutated APAs and 6 APAs without *KCNJ5* mutation. This is consistent with previous studies reporting sparse or no immune-cell infiltration within APAs compared to cortisol-producing adenoma [46]. These findings support the concept of tumor cells within the TME of APAs evading immune surveillance.

**Figure 5:**
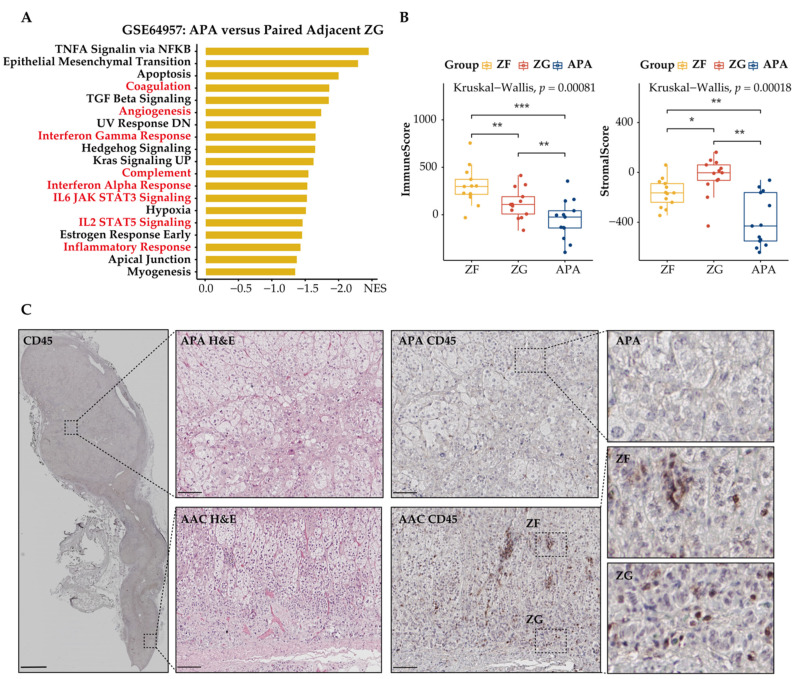
Spatial organization of tumor-infiltrating immune cells in APAs. Tumor-infiltrating immune cells in APAs were determined versus adjacent zona glomerulosa (ZG), and adjacent zona fasciculata (ZF). (**A**) GSEA showing MSigDB hallmark of downregulated differentially expressed genes in APAs versus adjacent ZG (GSE64957). Pathways in red indicate functions related to immune response. (**B**) ESTIMATE algorithm showing the distribution of ImmuneScore, StromalScore among APAs, adjacent ZG, and adjacent ZF. * *p* < 0.05, ** *p* < 0.01, *** *p* < 0.001 by paired Wilcox test. Kruskal–Wallis test was used between groups. (**C**) Immunohistochemistry of H&E and CD45 staining among APA, adjacent ZF, and adjacent ZG. Overview of CD45 image: scale bar 2 mm. Deep zoom images: scare bar 100 μm. APA, aldosterone-producing adenoma; ACC, adjacent adrenal cortex; GSEA, gene set enrichment analysis; NES, normalized enrichment score; ZG, zona glomerulosa; ZF, zona fasciculata.

We used a similar approach to assess tumor-infiltrating immune cells of APAs, paired adjacent zona fasciculata, and adjacent zona glomerulosa (Figure 5A–C). APA had fewer CD45^+^ immune cells compared with the adjacent zona glomerulosa and adjacent zona fasciculata, suggesting an immunosuppressive microenvironment at the local site of APA. CD45^+^ immune cells in the adjacent zona fasciculata showed higher levels compared to those in adjacent zona glomerulosa, indicating that fatty acid β-oxidation may not contribute to the immunosuppressive properties of APA.

### 3.5. Distinct Immune Microenvironment Landscapes in APAs vs. Paired Adjacent Zona Glomerulosa

**Figure 6:**
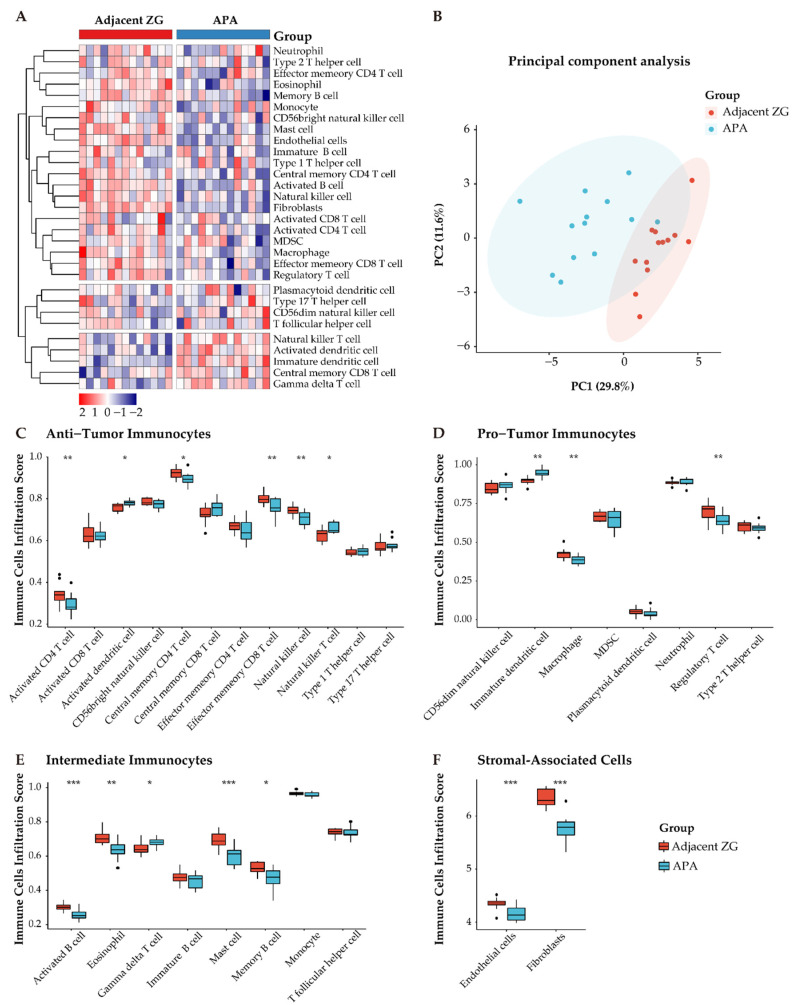
Distinct immune microenvironment landscapes in APA versus paired adjacent zona glomerulosa (ZG). (**A**) Heatmap of 28 tumor infiltration cells between APAs and adjacent ZG (GSE64957). MDSC, myeloid-derived suppressor cells. (**B**) Principal component analysis (PCA) of tumor-infiltrating cells. Two distinct groups were plotted in two-dimensional space: APA and adjacent ZG. PC, principal component. (**C**–**E**) Boxplot of the proportions of tumor microenvironment immune cells in (**A**) using the ssGSEA algorithm. (**F**) Boxplot of the proportions of TME stromal associated cells in (**A**) using MCP-counter algorithm. Box plots: scattered dots, immune score of the two subgroups; middle lines, median value; bottom and top of the boxes, 25th–75th percentiles. ns, not significant; * *p* < 0.05; ** *p* < 0.01; *** *p* < 0.001 by paired Wilcoxon test. APA, aldosterone-producing adenoma; ZG, zona glomerulosa.

To further explore differences in the composition of immune cells of APAs and paired adjacent zona glomerulosa, we performed ssGSEA using the MCP-counter algorithm, a method to profile fractions of immune cells by deconvolution of gene expression data, as shown in a heatmap (Figure 6A). Notably, principal component analysis showed two distinct clusters of tumor-infiltrating immune cells of the TME (APAs vs. paired adjacent zona glomerulosa) (Figure 6B). Further, we observed decreased anti-immune cells (e.g., activated and central memory CD4 T cells, effector memory CD8 T cells, and nature killer cells) and increased pro-immune cells (immature dendritic cells) (Figure 6C–F).

### 3.6. Functional Characterization of Immune-Related Differentially Expressed Genes in APAs

**Figure 7:**
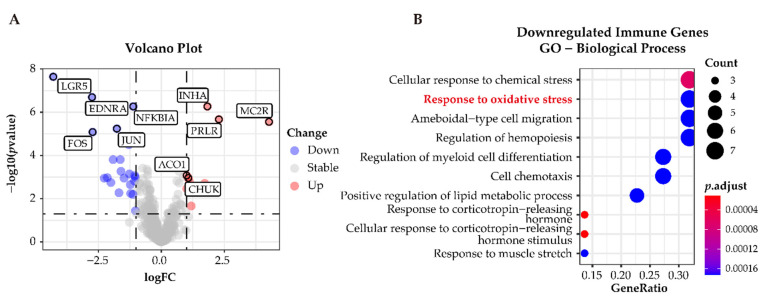
Functional characterization of immune-related differentially expressed genes in APAs versus paired adjacent zona glomerulosa (ZG). (**A**) Volcano Plot showing immune-differentially expressed genes (adjust *p*-value < 0.05 and |log_2_FC| > 1) in APA versus adjacent ZG (GSE64957). Each point represents a gene. Red and Blue dots represent upregulated and downregulated immune-differentially expressed genes, respectively. (**B**) GO analysis of downregulated immune-differentially expressed genes in (**A**). APA, aldosterone-producing adenoma; ZG, zona glomerulosa.

To classify immune-related genes, we intersected the whole dataset of differentially expressed genes with immune-related genes from ImmPort. A total of 31 differentially expressed immune-related genes were identified; 9 of these 31 were upregulated and the remaining 22 genes were downregulated in APAs versus paired adjacent zona glomerulosa (Figure 7A). GO analysis determined downregulated genes related to the immune response were enriched in the pathways related to “cellular response to oxidative stress” (Figure 7B), suggesting that oxidative stress may elicit an inflammatory response in the adjacent zona glomerulosa.

### 3.7. Enhanced Anti-Oxidative Response Pathways in APAs

**Figure 8:**
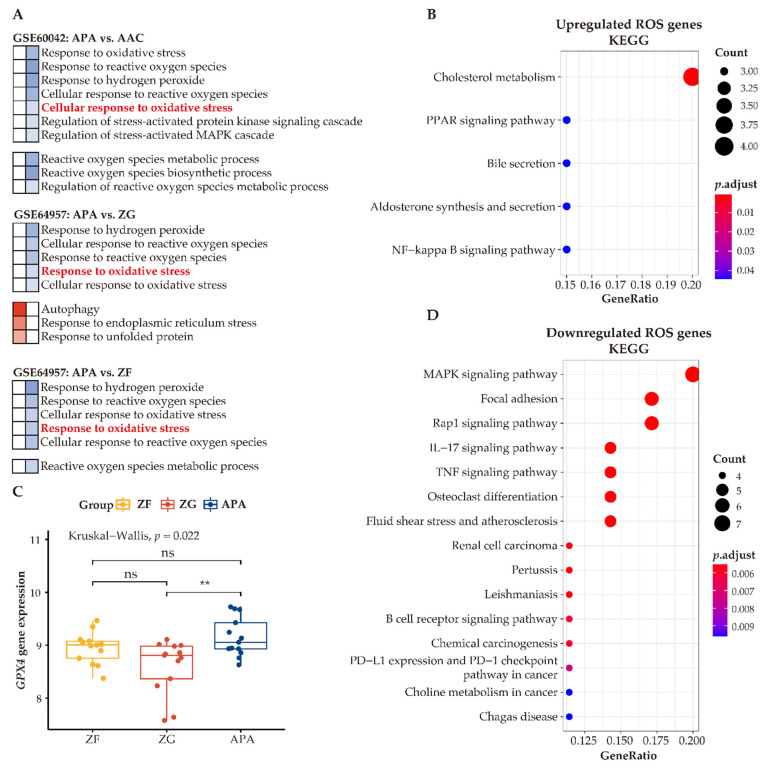
Enhanced antioxidative response pathways in APAs. (**A**) GO analysis of GSEA showing heatmap of ROS-related pathways. Red and blue color indicate upregulated and downregulated pathways ordered by normalized enrichment score in APAs versus AAC, adjacent ZG, and adjacent ZF, respectively. Normalized enrichment score >0 means upregulated pathways (Red), whereas normalized enrichment score <0 means downregulated pathways (Blue). Corresponding pathway enrichments are listed on the side. (**B**,**D**) indicate KEGG pathways of differentially expressed genes involved in ROS in APA versus adjacent ZG. Overexpressed ROS gene categories associated with metabolism pathways, whereas those downregulated ROS genes are associated with immune-response pathways. (**C**) The *GPX4* gene expression between APAs, adjacent ZG, and adjacent ZF. ** *p* < 0.01 by paired Wilcoxon test. Kruskal–Wallis test was used between groups. APA, aldosterone-producing adenoma; AAC, adjacent adrenal cortex; ZG, zona glomerulosa; ZF, zona fasciculata; ROS, reactive oxygen species.

Our analyses indicate that adjacent zona glomerulosa are challenged with increased oxidative stress compared to cells of APAs. To investigate the function of ROS within the context of APA cells, we compared differentially expressed genes functionally related to ROS in APAs vs. paired adjacent zona glomerulosa. We identified 22 upregulated ROS genes and 40 downregulated ROS genes. KEGG analysis of the upregulated ROS genes demonstrated upregulation of several metabolic pathways, including cholesterol metabolism, peroxisome proliferator-activated receptor (PPAR) signaling, aldosterone synthesis, and secretion (Figure 8B), suggesting that ROS may be involved in the regulation of metabolism and/or affected by the intermediates of metabolic alterations. In contrast, the most prominently altered processes of downregulated genes related to ROS were the inflammatory response pathways (Figure 8D). Collectively, the link of ROS with metabolism and the inflammatory response may suggest contributory biological mechanisms to the pathogenesis of APAs.

To address how APAs accommodate high ROS levels and ameliorate oxidative stress, we summarized ROS-related pathways between APAs versus paired adjacent adrenal cortex, APAs vs. paired adjacent zona glomerulosa, and APAs versus paired adjacent zona fasciculata. In general, all adjacent tissues showed higher enrichment of genes involved in ROS-related pathways such as “cellular response to oxidative stress” (Figure 8A). Therefore, we postulated that adrenocortical tumor cells may increase antioxidant properties to counteract metabolic stress. Accordingly, we determined upregulation of the unfolded protein response pathway in APAs (vs. adjacent zona glomerulosa) [47], which is an adaptive mechanism to relieve endoplasmic reticulum stress and restore cellular metabolic function, thereby promoting the survival of tumor cells. In addition, autophagy, a cellular stress-response mechanism [48] that recycles key metabolites under metabolic stress and promotes cellular adaptation to oxidative stress, was also enhanced in APAs. In addition, glutathione peroxidase 4 (GPX4) mRNA levels, a key enzyme for antioxidant defense, were higher in APAs among groups (Figure 8C). Together, these mechanisms ensure an efficient alleviation of oxidative stress that APA cells encounter during excess aldosterone production and abnormal proliferation.

## 4. Discussion

In this study we used advanced bioinformatics tools to comprehensively evaluate the TME of APAs. We demonstrated that metabolic reprogramming towards fatty acid β-oxidation and glycolysis is a general feature of APAs that may provide a metabolically favorable environment for tumor growth. Furthermore, we showed an immunosuppressive microenvironment in APA and diverse cellular components of the TME (e.g., immune and stromal cells) between APAs and the adjacent zona glomerulosa. 

We showed that lipid metabolism is highly associated with APA tumorigenesis. Metabolism reprogramming enables tumor cells to sustain ATP generation for cell growth, division, and survival. Notably, dysregulation of lipid metabolism has been demonstrated as a prominent metabolic alteration in cancers [49]. In particular, increased β-oxidation of stored lipids provides a source of NAPDH and ATP—approximately six times that of oxidation of carbohydrates—through the oxidation of acetyl-CoA by the tricarboxylic acid (TCA) cycle, thereby facilitating tumor progression [50]. Furthermore, NAPDH is critical for two steps in steroidogenesis pathways in the adrenal gland: (1) the rate-limiting step for the conversion of cholesterol to pregnenolone catalyzed by CYP11A1 and (2) the conversion of deoxycorticosterone to aldosterone catalyzed by CYP11B2 [51,52]. Thus, elevated fatty acid β-oxidation in APAs may stimulate aldosterone synthesis by the high metabolic activity of NAPDH generation in mitochondria. In addition, our bioinformatics studies implied that changes in lipid metabolism in APAs are regulated by PPARα. Previous studies reported that transcriptional activation of PPARα regulates β-oxidation in tissues displaying high energy consumption [53,54]. Consistent with our findings, a previous study demonstrated that fenofibrate, a PPARα agonist, increased angiotensin II-independent *CYP11B2* mRNA expression and aldosterone production in human adrenocortical carcinoma H295R cells, whereas a peroxisome proliferator-activated receptor-γ (PPARγ) agonist either had no effect or reduced aldosterone secretion [55]. These data indicate that high activity of fatty acid β-oxidation induced by PPARα signaling may be crucial for excess aldosterone production.

Further to a role for fatty acid β-oxidation in APA pathogenesis, we reported that glycolysis metabolism may also play a key role, especially in *KCNJ5*-mutated APAs. This latter finding is consistent with a mass spectrometry imaging study that identified the activation of glycolysis pathways in APAs as well as in a subgroup of aldosterone-producing micronodules [56] (previously known as aldosterone-producing cell clusters [57]). In contrast, a high-resolution mass spectrometry imaging map of the normal human adrenal gland reported that glycolysis/gluconeogenesis was found to be significantly increased in the medulla [58]. This finding is consistent with the concept that a metabolic switch to glycolysis confers a selective advantage for tumor growth. However, the underlying mechanism of such metabolic phenotypes is unclear. Of potential interest, lactate infusion alone, or in combination with angiotensin II, results in increased aldosterone secretion from rat zona glomerulosa cells [59]. Therefore, the precise relationship between glycolysis and its metabolites and aldosterone production merits further investigation. 

Our study showed the low tumor infiltrating CD45^+^ lymphocyte density in APAs. Such a scenario is in line with previous reports showing sparse immune cell infiltration using hematoxylin-eosin staining in APAs relative to cortisol-producing adenomas [46]. This alteration in the TME may be accounted for by competition for nutrients in the TME, and glycolysis intermediates forming an acidic microenvironment and thereby suppressing immune activation [27,60]. Another potential mechanism is the promotion of immunosurveillance evasion by activated PPAR signaling [61]. PPARα exerts anti-inflammatory activity, for example, and PPARα agonists mediate a variety of effects on the immune response to reverse acute and chronic liver inflammation [54]. Consistently, PPARα-deficient aged mice show a pro-inflammatory phenotype [62]. In addition, the PPARα agonist fenofibrate caused a reduction in blood pressure, especially in salt-sensitive hypertensive subjects [63], suggesting that PPARα may play a role in regulation of renin–angiotensin–aldosterone system activity, and influence aldosterone secretion. This discrepancy may be explained, however, by its dual influence on systemic and local tumor levels. Furthermore, through expression–signature-based approaches, we observed low effector memory CD8 T cell infiltration in APAs, which is in agreement with a previous study that demonstrated decreased viability of effector T cells in a glucose-restricted medium in vitro [64]. These data suggest that metabolic reprogramming towards glycolysis in APA may impose a hypoglycemic environment, restrict glucose uptake by immune cells and therefore hamper their function. In addition, a previous study showed that the distribution of mast cells is more frequently visualized in the adjacent cortex of APAs [11], consistent with the high mast cell infiltration in adjacent zona glomerulosa relative to APAs in our study.

Active steroidogenesis has been implicated in contributing to high ROS production and oxidative stress, triggering cell death [65]. Our analyses demonstrated that the adjacent cortex is challenged with increased oxidative stress compared to APAs, regions of high steroidogenesis. Our finding of enhanced fatty acid β-oxidation in APAs may explain this apparent paradox because it provides NAPDH, which may counteract ROS toxicity from metabolic stress [66]. Furthermore, fatty acid β-oxidation is increased in zona fasciculata relative to paired zona glomerulosa, likely because glucocorticoid production in normal zona fasciculata produces significantly more cellular ROS than from aldosterone synthesis in normal zona glomerulosa due to 40% of “leaky” electrons in the P450c11β (CYP11B1) system [67]. This would require elevated fatty acid β-oxidation for protection of adrenocortical zona fasciculata cells from ROS. In addition, previous work demonstrated that enhanced glycolysis can combat oxidative stress via increasing glutathione metabolism and maintaining redox balance [68], a potential factor contributing to the larger tumor diameter in APAs with *KCNJ5* mutations. Additionally, our data showed an increased antioxidant response via an enhanced unfolding protein response and autophagy [47,48], suggesting that multiple mechanisms participate in the detoxification of ROS and aid adrenocortical tumor cell survival. 

It has been reported that adrenocortical cells are sensitive to ferroptosis-triggering agents, such as RSL3, which can inhibit GPX4 activity [17,45]. Indeed, our data showed elevated *GPX4* expression in APAs compared with adjacent zona glomerulosa. Adrenocortical tumor cells must boost their antioxidant capacity to counteract the lipid peroxidation and oxidative stress induced by steroidogenesis to suppress cell death by ferroptosis. Although studies into the molecular mechanisms underlying the adaptation of APA cells to high ROS generation in the local tumor site to circumvent ferroptosis are not well defined, lipid metabolism likely participates, and in particular fatty acid β-oxidation. This hypothesis is partially supported by Kagan et al., who showed that etomoxir, an inhibitor of mitochondrial fatty acid β-oxidation, enhanced RSL3-induced ferroptosis in mouse embryonic fibroblasts [69]. Further, PPARα activator can reduce lipid peroxidation [70]. Considering the known association of fatty acid β-oxidation with PPARα, PPARα activation may feasibly regulate the susceptibility of adrenocortical cells to lipid peroxidation, a key characteristic of ferroptosis, through β-oxidation. Furthermore, we observed increased *SCD* and *GCLC* mRNA expression in APAs. Two key enzymes catalyze monounsaturated fatty acid synthesis and biosynthesis of glutathione, which protect tumor cells against ferroptosis inducers [71,72]. These exemplify the central hub role of metabolism reprogramming to adapt to metabolic stress.

This study reports a potential role of fatty acid oxidation in supporting adrenocortical cell growth; however, further research is required to delineate the precise mechanisms involved. Our study had several limitations, including the absence of protein-level data corresponding to identified differentially expressed genes related to lipid metabolism with a potential role in APA pathophysiology. In addition, we did not fully characterize the role of oxidative stress (lipid peroxidation) in APA tissues, which warrants further investigation. 

## 5. Conclusions

It is challenging to evaluate the tumorigenic landscape of APAs using experimental methods. As such, it is still unclear how APA tumor adrenocortical cells maintain hypersecretion and cell proliferation despite a nutrient-deprived environment. We address this knowledge gap by shedding light on the energy metabolism and tumor immune microenvironment. Our analyses reveal that metabolic reprogramming involving a switch to fatty acid β-oxidation and glycolysis may stimulate aldosterone production, disturb the TME, mitigate oxidative stress, and support tumor cell survival. Therefore, we highlight metabolic reprogramming as a putative novel mechanism in APA pathophysiology.

## Data Availability

The publicly archived datasets presented in this study can be accessible through GEO series accession number GSE64957 and GSE60042.

## Abbreviations

**Abbreviation**	**Definition**
PA	Primary aldosteronism
APA	Aldosterone-producing adenoma
AAC	Adjacent adrenal cortex
ZG	Zona glomerulosa
ZF	Zona fasciculata
TME	Tumor microenvironment
ROS	Reactive oxygen species
GO	Gene Ontology
KEGG	Kyoto Encyclopedia of Genes and Genomes
GSEA	Gene set enrichment analysis
MCP-COUNTER	Microenvironment cell populations-counter
ESTIMATE	Estimation of Stromal and Immune cells in Malignant Tumors using Expression data
GCLC	Glutamate-cysteine ligase catalytic subunit
SCD	Stearoyl-CoA desaturase
GPX4	Glutathione peroxidase 4
ATP	Adenosine triphosphate
NADPH	Nicotinamide adenine dinucleotide phosphate
TME	Tumor microenvironment
